# Enhancing Osteoblastic Cell Cultures with Gelatin Methacryloyl, Bovine Lactoferrin, and Bioactive Mesoporous Glass Scaffolds Loaded with Distinct Parsley Extracts

**DOI:** 10.3390/biom13121764

**Published:** 2023-12-09

**Authors:** Laura Isabel Arias-Rodríguez, Jesús L. Pablos, María Vallet-Regí, Martha A. Rodríguez-Mendiola, Carlos Arias-Castro, Sandra Sánchez-Salcedo, Antonio J. Salinas

**Affiliations:** 1 Plant Biotechnology Laboratory, Instrumental Analysis Laboratory and Plant Biochemistry Laboratory of the National Technological Institute of Mexico Campus Tlajomulco, 10th km Tlajomulco Highway, Southern Metropolitan Circuit, Tlajomulco de Zúñiga 45640, Jalisco, Mexico; lauraardz@gmail.com (L.I.A.-R.); martha.rm@tlajomulco.tecnm.mx (M.A.R.-M.); carlos.ac@tlajomulco.tecnm.mx (C.A.-C.); 2 Department of Chemistry in Pharmaceutical Sciences, Faculty of Pharmacy, Complutense University of Madrid (UCM).12 de Octubre Hospital Research Institute, Imas12, 28040 Madrid, Spain; jesuslpa@ucm.es (J.L.P.); vallet@ucm.es (M.V.-R.); 3 Networking Research Center on Bioengineering, Biomaterials and Nanomedicine, CIBER-BBN, 28040 Madrid, Spain

**Keywords:** mesoporous bioactive glasses, bovine lactoferrin, embryogenic cultures, parsley, scaffolds

## Abstract

The increasing interest in innovative solutions for addressing bone defects has driven research into the use of Bioactive Mesoporous Glasses (MBGs). These materials, distinguished by their well-ordered mesoporous structure, possess the capability to accommodate plant extracts with well-established osteogenic properties, including bovine lactoferrin (bLF), as part of their 3D scaffold composition. This harmonizes seamlessly with the ongoing advancements in the field of biomedicine. In this study, we fabricated 3D scaffolds utilizing MBGs loaded with extracts from parsley leaves (PL) and embryogenic cultures (EC), rich in bioactive compounds such as apigenin and kaempferol, which hold potential benefits for bone metabolism. Gelatin Methacryloyl (GelMa) served as the polymer, and bLF was included in the formulation. Cytocompatibility, Runx2 gene expression, ALP enzyme activity, and biomineralization were assessed in preosteoblastic MC3T3-E1 cell cultures. MBGs effectively integrated PL and EC extracts with loadings between 22.6 ± 0.1 and 43.6 ± 0.3 µM for PL and 26.3 ± 0.3 and 46.8 ± 0.4 µM for EC, ensuring cell viability through a release percentage between 28.3% and 59.9%. The incorporation of bLF in the 3D scaffold formulation showed significant differences compared to the control in all assays, even at concentrations below 0.2 µM. Combinations, especially PL + bLF at 0.19 µM, demonstrated additive potential, with superior biomineralization compared to EC. In summary, this study highlights the effectiveness of MBGs in incorporating PL and EC extracts, along with bLF, into 3D scaffolds. The results underscore cytocompatibility, osteogenic activity, and biomineralization, offering exciting potential for future in vivo applications.

## 1. Introduction

Regeneration is the process of replacing lost specialized tissue with specialized cells, and is mainly limited to a few human tissues, such as bone. Bone grafts are classified as autografts (from the recipient), allografts (from donors of the same species), and xenografts (from a different species) [1]. While autografts have traditionally been the gold standard for reconstructing bone defects, donor site morbidity remains a significant limitation. To address these challenges, there is growing interest in the field of bone tissue engineering (BTE) in bioactive compounds with therapeutic and preventive potential against bone resorption. Additionally, there is a focus on biomimetic strategies to guide the establishment and growth of osteogenic cells and growth factors in a well-defined three-dimensional environment [2].

Since 2001, research into ordered mesoporous silica-based materials has been aimed at their potential use in biomedical applications. Among these materials, Bioactive Mesoporous Glasses (MBGs) have gained significance in bone regenerative medicine due to their SiO_2_-CaO-P_2_O_5_ system. CaO is an essential component, while P_2_O_5_ is recommended for bioactivity and the generation of hydroxyapatite layers. Their textural properties allow them to host and release biologically active substances, functioning as drug delivery systems [2]. MBGs powders represent optimal candidates for crafting bone scaffolds—artificial structures designed to offer transient mechanical and physiological support for in vitro tissue regeneration and in vivo implantation. Furthermore, integrating MBGs powders into polymers to create composites holds promise for significantly enhancing the material’s bioactivity. These compounds may be applied as scaffolds in the context of bone healing, yielding multifunctional materials endowed with exceptional properties [3,4]. Using 3D printing, these materials can mimic bone tissue, containing large pores (channels) and macropores to facilitate angiogenesis and cell interaction, along with nanopores in the range of 2 to 10 nm, which are suitable for loading known biologically active substances, such as those with osteogenic, angiogenic, or antibacterial properties [2,5,6].

Currently, there is an ongoing research focused on incorporating these materials with various therapeutic ions, such as strontium, copper, and zinc, among others [7,8,9,10]. Furthermore, a thorough examination has been conducted on the loading and release of well-recognized therapeutic drugs for the treatment of bone conditions, including alendronate, simvastatin, and dexamethasone [11,12,13]. Additionally, there is an active exploration into growth factors, bioactive molecules such as peptides and proteins, non-viral genetic elements (DNA, RNA), and plant-derived drugs like icariin and curcumin [14,15]. The use of bioactive compounds derived from plant extracts has attracted attention due to their favorable attributes, which could potentially enhance therapeutic efficacy and reduce dosage requirements, thereby minimizing the risk of toxicity and side effects associated with conventional pharmacological agents [16]. Some reported examples of extracts that have demonstrated osteogenic effects when integrated with bioactive glasses include Curcuma longa, Yunnan Baiyao ointments, Neem, cranberry, Coumarin (*Tonka bean*), Daidzein, Icariin (*Epimedium sagittatum*), and Lawsone (*Lawsonia inermis*) [17,18].

While it is known that extracts are complex mixtures of compounds, it is important to highlight that recent studies in the last few years have described various research endeavors involving plant extracts and isolated flavonoids, allowing for the identification of compounds with a greater tendency to impact bone metabolism. Notably, compounds such as apigenin, quercetin, rutin, and kaempferol have specific regulatory effects related mainly to osteoblast differentiation through the increased alkaline phosphatase (ALP) activity, enhancement of osteocalcin (OCN) gene expression, upregulation of collagen-1A1 (Col-1A1), and the promotion of mitogen-activated protein kinase (MAPK) activity, leading to the expression and activation of Runx2 (essential for osteoblast differentiation) [19,20].

Moreover, *P. crispum*, commonly known as parsley, boasts an abundance of bioactive compounds, primarily concentrated in its leaves, with apigenin emerging as the most prominent among them. This high apigenin content renders parsley an appealing subject for rigorous scientific inquiry [20,21]. Extracts sourced from parsley leaves have demonstrated a diverse array of therapeutic attributes, acting as a diuretic, anti-inflammatory, antioxidant, antiplatelet, anti-urolithiasis, anticancer, and anti-aging agent. Furthermore, parsley extracts have been linked to a diminished risk of cardiovascular disease [22]. In addition, recent investigations involving embryogenic cultures of parsley have unveiled significant variances in flavonoid production, especially in the case of apigenin and kaempferol, surpassing levels observed in conventional cultures [16]. Consequently, both parsley leaf (PL) and embryogenic cultures (EC) of parsley emerge as captivating alternatives for exploration when integrated into MBGs scaffolds.

Conversely, bovine lactoferrin (bLF), a glycoprotein abundantly present in milk serum, has garnered significant attention in recent years as a potent stimulator of tissue development, particularly in the context of bone tissue. This prominence is attributed to its noteworthy influence on the differentiation of osteoblastic and chondroblast cells (those responsible for cartilage formation). Within the cellular milieu, bLF elevates the expression of pivotal factors, including Runx2 and Sox9 (a key regulator in cartilage development), and augments the presence of ALP and OC, both of which are essential proteins for bone formation and remodeling and are produced by osteoblastic cells [23]. Furthermore, bLF has demonstrated its capacity to interact with flavonoids, leading to additive or synergistic effects of substantial medicinal significance [24,25,26].

Therefore, this study aims to generate pioneering and interdisciplinary findings. It involves the integration of extracts primarily enriched with apigenin and sourced from parsley leaves, as well as extracts obtained from a novel source of bioactive components, specifically from in vitro parsley-habituated embryogenic cultures—all with the aim of incorporating them into MBGs. The main objective is to assess the in vitro effects of two different parsley extract-enriched MBGs on the viability and functionality of Mouse MC3T3-E1 preosteoblastic cells within 3D-printed scaffolds made from Gelatin Methacryloyl (GelMa)/MBGs. This innovative approach involves the incorporation of bovine lactoferrin (bLF) to enhance the scaffolds’ properties. The unique combination of 3D-printed scaffolds, bovine lactoferrin, and parsley extract-loaded MBGs not only enriches the scaffold composition but also opens a pioneering pathway for prospective in vitro studies. This novel strategy holds great promise for advancing the frontier of in vitro research in bone regeneration.

## 2. Materials and Methods

### 2.1. Parsley Leaf (PL) and Embryogenic Culture (EC) Material

To prepare the plant leaves, 1 kg of parsley (*P. crispum*) was acquired from a local supermarket in Santa Anita, Tlaquepaque, Jalisco, Mexico. The leaves were meticulously separated, washed, disinfected, and laid out on absorbent paper for 4 h. Subsequently, they were frozen at −20 °C for 24 h to facilitate lyophilization and were stored in a desiccator dryer for future extraction.

Embryogenic cultures were established following the procedure outlined by Arias-Rodríguez et al. [16]. At intervals of 21 days during incubation, the cultures were harvested, frozen at −20 °C for 24 h to facilitate lyophilization, and then stored in empty desiccators for subsequent extraction.

### 2.2. Parsley Leaf and Embryogenic Cell Culture Extractions

Fifteen grams each of lyophilized leaf and embryogenic culture samples were added to boiling distilled water at a ratio of 1:15 (*w*/*v*). The entire process was conducted in triplicate, following the procedure established by Arias-Rodríguez et al. [16], to obtain hydrolyzed extracts.

### 2.3. HPLC Extract Analysis

Standards of apigenin (A) and kaempferol (K) with 95% purity were procured from Sigma-Aldrich, Merck, Mexico. Calibration curves were established by dissolving these standards in HPLC-grade methanol at six different concentrations (μg/mL): 0.125, 0.25, 0.50, 1.00, 2.00, and 10.00. Subsequently, hydrolyzed extracts were diluted at a ratio of 1:300 (*v*/*v*) with the same methanol.

Identification and quantification were conducted based on the procedure outlined by Arias-Rodríguez et al. [16], utilizing a Thermo Fischer^®^ HPLC system. The system comprised a High-Performance Anionic Exchange Chromatography-Pulsed Amperometric Detector coupled to a diode array detector (DAD), with a Gemini-NX C18 Column (150 mm × 4.60 mm) from Phenomenex^®^. Elution was performed in a gradient system with (A) water acidified with acetic acid 97:3 and (B) acetonitrile, starting with 10% B and increasing to 90% over 2 to 15 min. The injection volume was 15 μL with a flow rate of 1 mL/min, and the column temperature was maintained at 30 °C. Two wavelengths (337 and 363 nm) were employed for flavonoid detection.

### 2.4. Synthesis and Characterization of Bioactive Mesoporous Glasses (MBGs)

Synthesis was conducted employing the evaporation-induced self-assembly (EISA) method, following the procedure previously published by Heras et al. (2020), with some modifications. All the reagents were procured from Sigma-Aldrich, St. Louis, MO, USA.

In a beaker, 4.5 g of surfactant F127, 85 mL of ethanol (99.98%), and 1.2 mL of 0.5 N HNO_3_ were mixed for 1 h at 250 rpm and 30 °C, with the container sealed using Parafilm^®^ to prevent solvent evaporation. Subsequently, 8.9 mL of tetraethyl orthosilicate (TEOS), 0.71 mL of triethyl phosphate (TEP), and 1.10 g of Ca (NO_3_)_2_·4H_2_O were added as SiO_2_-P_2_O_5_-CaO sources. A 1 h waiting period was observed following the addition of each reagent. The mixture was stirred under the same conditions mentioned earlier for 18 h.

The resulting solution was transferred to Petri dishes (25 mL/dish) to facilitate ethanol evaporation at 30 °C. Transparent membranes were obtained after 7 to 10 days, which were subsequently cut into pieces and placed in crucibles for calcination at 700 °C for 6 h with a temperature ramp of 5 °C/min. The calcined MBGs were ground using a glass mortar and passed through a 40 µm sieve to obtain homogeneous powder.

Sample physicochemical characteristics of the material were characterized by small-angle X-ray diffraction (SA-XRD) utilizing an X’Pert-MPD system (Eindhoven, Netherlands) equipped with Cu Kα radiation in the range of 0.6° to 8° 2θ, and a Fourier-transform infrared spectroscopy (FTIR) was conducted using a Thermo Scientific Nicolet iS10 spectrometer (Waltham, MA, USA) equipped with an attenuated total reflection (ATR) SMART Golden Gate accessory.

Furthermore, textural properties were determined by nitrogen adsorption, employing a Micromeritics 3 Flex instrument (Norcross, GA, USA). This allowed for the calculation of the specific surface area (S_BET_) using the Brunauer–Emmett–Teller (BET) method and pore size distributions using the Barret–Joyner–Halenda (BJH) method. Ordered mesoporosity was analyzed through transmission electron microscopy (TEM) using a JEM-2100 JEOL microscope operating at 200 kV (Tokyo, Japan). Composition was determined via energy-dispersive X-ray spectroscopy (EDS) with an Oxford LINK EDS analyzer coupled to the TEM microscope.

### 2.5. Synthesis and Degree of Substitution Analysis of Methacrylated Gelatin (GelMa)

Synthesis was conducted according to the methodology reported by Pablos et al. [27], with some modifications in temperature. Gelatin (Type A, porcine skin) and Methacrylic Anhydride (MA) were obtained from Sigma-Aldrich, USA, while 10× Phosphate-Buffered Saline (PBS) was purchased from Gibco (Thermo Fisher Scientific, Grand Island, New York, NY, USA).

The procedure began by dissolving 10 g of gelatin in 100 mL of 1× PBS at 60 °C with continuous agitation. Once dissolved, 8 mL of MA (0.8 mL/g) was added at a rate of 0.5 mL/min. The reaction was vigorously stirred for 3 h. Subsequently, 50 mL of preheated 1× PBS at 50 °C was added. The resulting GelMa solution was dialyzed in a Milli-Q water solution for 7 days at 30 °C to remove Methacrylic Anhydride and other impurities, using a 12–14 kDa dialysis membrane. The dialyzed solution was frozen at −20 °C and then lyophilized.

To quantify the amount of methacryloyl groups in GelMa, analyses were performed using proton nuclear magnetic resonance (^1^H-NMR) (400 MHz Varian), following the protocol described by Shirahama (2018). In summary, 50 mg of a lyophilized GelMa sample was dissolved in 1 mL of deuterium oxide (D_2_O) at 30 °C, along with porcine skin gelatin.

For quantification, the area of the methylene proton peak of lysine at around 2.9 ppm was used in each spectrum obtained for gelatin and GelMa samples, using, as reference, the peak area of aromatic acids (phenylalanine signal at 7.3 ppm) in the gelatin and GelMa samples in each spectrum (peak at 7.4 ppm). Subsequently, the amount of methacryloyl groups (DM) was calculated using the following Equation (1) [27]:

(1) $$DM\left(\%\right)=\left(1-\frac{Area\;of\;lysine\;methylene\;of\;GelMa}{Area\;of\;lysine\;methylene\;of\;Gelatin}\right)\times 100$$

### 2.6. In Vitro Cytocompatibility Assays of Apigenin, Kaempferol, Extracts, and bLF in Pre-Osteoblastic MC3T3-E1 Cells

For this assay, three replicates were arranged for the control and three for each previously sterilized sample. Mouse MC3T3-E1 preosteoblastic cells (subclone 4, CRL-2593; ATCC, Manassas, VA, USA) were employed. The cells were cultured in supplemented growth medium, α-MEM, with 10% fetal bovine serum (FBS, Gibco, Thermo Fisher Scientific, Wilmington, DE, USA), 1% penicillin-streptomycin, and 5 mM L-glutamine (Gibco, Thermo Fisher Scientific, Wilmington, DE, USA), under 5% CO_2_ saturation at 37 °C. The cells were washed with pH 7.4 PBS and harvested using 0.25% trypsin-EDTA (Gibco, Thermo Fisher Scientific, Wilmington, DE, USA). The cell suspension was centrifuged for 7 min at 1200 rpm, and the resulting cells were suspended in a fresh supplemented medium. Cell counting was performed to achieve a seeding density of 2·10^3^ cells/mL per well, to which 900 µL of the medium and 100 µL of the standards or samples were added.

The concentrations employed in the extracts were determined based on the quantification of apigenin in prior HPLC analyses. Both the standards and extracts were prepared using 99.9% EtOH with a final concentration of DMSO under 10%. Bovine lactoferrin (bLF) was dissolved in Milli-Q water and subsequently filtered through a 45 µm Whatman filter.

The concentrations employed (in µM) are delineated as follows:

(A) Apigenin and kaempferol (1:1 *v*/*v*): 1, 5, and 10.

(B) bLF: 0.12, 1.2, and 12.

These selections were based on concentrations previously employed and documented in in vitro assays [28,29,30,31,32,33].

(C) Parsley leaves and embryogenic cultures: 1, 5, and 10.

Concentrations were based on the quantity of apigenin previously detected in each extract through HPLC [15].

### 2.7. Loading and Release of Extracts in Photopolymerized GelMa/MBGs Discs

#### 2.7.1. Loading of Apigenin, Kaempferol, and Extracts in MBGs and Processing into GelMa/MBGs Discs

The loading procedure followed the methodology and material’s release behavior described by Heras et al. [7] with some modifications. Concentrations were prepared through dilution using 99.98% EtOH, and these concentrations were chosen based on previous in vitro results. Standards loadings were conducted at concentrations of 50 and 100 µM. For the extracts, the concentrations were based on the quantity of apigenin previously detected in each extract through HPLC.

The MBGs/extract ratio was 100 mg/3 mL, and they were continuously stirred for 24 h at 100 rpm at room temperature. Subsequently, the material was rinsed and centrifuged three times with 99.98% EtOH and allowed to evaporate in an oven at 30 °C for two days in the dark.

Prior to the preparation of discs, the loading of MBGs was quantified through TGA analysis, and the reduction in surface area was determined through a nitrogen adsorption measurement (S_BET_).

The paste for making the discs was prepared by dissolving 2.6 mg of the photoinitiator Irgacure 2959 in 666 µL of Milli-Q water with vigorous stirring at 50 °C. Then, 133 mg of GelMa (0.8 mg/g) was gradually dissolved. In order to prevent interference in the quantification of apigenin release from the discs in this experiment, the addition of bLF, along with GelMa, was omitted in this assay. When the solution became transparent without any lumps, 400 mg of loaded MBGs (extracts and standards) were added to create a thick paste. This paste was transferred to a transparent polyethylene mold and exposed to UV light using a high-intensity Blak-Ray^®^ B-100AP UV lamp (Upland, CA, USA), with a wavelength of 365 nm for 30 min on each side.

Upon completing the photopolymerization time, the discs were transferred to sterile 12-well polystyrene plates and sterilized under UV light for 24 h before the in vitro assay.

#### 2.7.2. High-Performance Liquid Chromatography (HPLC) Analysis of Apigenin, Kaempferol, and Extract Release

For this assay, a Thermo Fischer^®^ HPLC system comprising a high-performance anion exchange chromatography with pulsed amperometric detection coupled to a diode array detector (DAD) was employed. A Phenomenex^®^ Gemini-NX C18 column (150 mm × 4.60 mm) was used. Elution was carried out using a gradient system composed of the following solvents: (A) 97:3 water acidified with acetic acid and (B) acetonitrile. The gradient commenced with 10% B and was increased to 90% B over a period of 2 to 15 min. An injection volume of 15 μL was used, with a flow rate of 1 mL/min. The column temperature was maintained at 30 °C, and two wavelengths (337 and 363 nm) were employed for flavonoid detection.

Three replicates of each sample (apigenin, kaempferol, parsley leaf (PL), and embryogenic culture (EC) extracts) were prepared in sterile 12-well polystyrene plates. To each sample, 1.5 mL of sterile Milli-Q water was added, and they were maintained at 37 °C with constant agitation at 80 rpm. Supernatants were collected every 24 h for 7 days. Each supernatant was frozen at −20 °C, then lyophilized and suspended in 500 µL of HPLC-grade MeOH, followed by sonication for 5 min. Subsequently, the samples were centrifuged and further diluted at a 1:60 ratio with HPLC-grade MeOH for quantification. Calibration curves using 95% apigenin and kaempferol standards (Sigma-Aldrich, Darmstad, Germany) were established in HPLC-grade MeOH at six concentrations (µg/mL): 0.125, 0.25, 0.50, 1.00, 2.00, and 10.00. The quantification was performed using the HPLC system mentioned previously.

### 2.8. In Vitro Assays with Preosteoblastic MC3T3-E1 Cells Using Three-Dimensional GelMa/MBGs Scaffolds Loaded with Extracts and bLF

#### 2.8.1. The 3D Printing Process for Meso-Macroporous GelMa/MBGs Scaffolds

For the fabrication of three-dimensional scaffolds, a REGE4LIFE printer from the Spanish brand Regemat3D^®^ was utilized, along with the REGEMAT 3D Designer software. The printing process employed straight metallic nozzles with a diameter of 0.84 nm and a 5 mL plastic syringe.

The paste preparation followed the procedure outlined in Section 2.7.1, with modifications regarding the quantities used and the incorporation of bLf. This included 2.08 mL of Milli-Q water in which bLF was dissolved using two concentrations (0.094 and 0.19 µM), 8 mg of the photoinitiator Irgacure 2959, 400 mg of GelMa, and 1.2 g of MBGs. The printed scaffolds were irradiated in a prefabricated chamber using a high-intensity UV lamp, Blak-Ray^®^ B-100AP (Upland, CA, USA), operating at 230 V at 50 Hz with a current of 2.0 Amps with a wavelength of 365 nm for 30 min on each side, aiming to perform photo-initiated radical polymerization.

The parameters established for scaffold development included a syringe temperature of 40 °C, a flow rate of 1.0 mm/s, and a scaffold height and diameter of 7 × 10 mm^2^, with a layer height of 0.4 mm. Additionally, it was essential to maintain a platform temperature of 23 °C.

#### 2.8.2. Characterization of Meso-Macroporous 3D GelMa/MBGs Scaffolds

The analysis of the macroporosity of the scaffolds and their chemical composition was conducted using a scanning electron microscope JEOL JSM-644 (Tokyo, Japan). SEM micrographs were captured from both the pristine scaffold surface and a fractured scaffold, both before and after the in vitro study, in order to elucidate the morphology changes following compound release. Furthermore, an analysis of the outer layer was performed using Fourier-transform infrared spectroscopy (FTIR), and the textural properties were assessed through nitrogen adsorption (S_BET_).

#### 2.8.3. In Vitro Proliferation Assay and Cytocompatibility Assay of Meso-Macroporous 3D GelMa/MBGs Scaffolds Loaded with bLF and Extracts

Cell cultures were obtained using the same methodology described in Section 2.6. For this assay, three replicates were set up for the control and three for the previously sterilized samples. The scaffolds were pre-hydrated with a fresh medium and incubated with agitation for 1 h prior to cell seeding. Subsequently, cell seeding was carried out at a concentration of 1.5·10^6^ cells/mL on each scaffold with a 5 min interval (25 µL/5 min) to allow cells to settle on the surface. Once the seeding was completed, 2 mL of medium was added. The cytocompatibility of the material was assessed through cell proliferation on days 1, 3, and 7. The Alamar Blue methodology (Invitrogen, Thermo Fisher Scientific) was employed following the manufacturer’s instructions. The culture medium was replaced with 2 mL of Alamar Blue dilution, and the cells were incubated for 3 h at 37 °C and 5% CO_2_. After this incubation, the resulting product was collected for fluorescence analysis, which was measured at excitation and emission wavelengths of 560 and 590 nm, respectively, using a BioTek Synergy 4 spectrophotometer (Winooski, VT, USA).

The culture medium used on day 7 was employed for the lactate dehydrogenase (LDH) assay (Spinreact, Girona, Spain) following the manufacturer’s instructions. The mixture of medium and reagent was measured using quartz cells at a temperature of 37 °C every 60 s for 4 min at 340 nm in a Helios zeta UV-Vis spectrophotometer.

#### 2.8.4. Morphological Studies via Confocal Laser Scanning Microscopy

Cell morphology was studied in the scaffolds after 7 days of incubation using fluorescence microscopy with an OLYMPUS FV1200 confocal scanning microscope (OLYMPUS, Tokyo, Japan) equipped with a 60× Fluor water immersion objective (NA = 1). Prior to analysis, the medium was removed, and the scaffolds were washed twice with PBS. Subsequently, cells were fixed with 4% (*w*/*v*) paraformaldehyde containing 1% sucrose at 37 °C for 20 min. Afterward, they were washed with PBS and permeabilized with 0.5% Triton ×100 (Sigma-Aldrich), followed by a PBS rinse. Staining was performed using 10 µL of Phalloidin-ATTO 565 (diluted 1:40) and incubated at 37 °C for 20 min. Finally, the samples were rinsed, and 1 mL of Fluoroshield^®^ with DAPI (1:1000, Sigma Aldrich, St. Louis, MO, USA) was added, then stored in the dark until analysis. DAPI and Atto 565-phalloidin stainings appeared in blue and red, respectively. Micrographs were acquired using FV10-ASW software (version 4.2, Waltham, MA, USA) to construct a single 2D image, which was converted into a TIF image file using multiple images obtained from each section along the *Z*-axis through an algorithm that displays the maximum pixel value of each Z-section for every 1 μm.

#### 2.8.5. Real Time PCR Assay

To evaluate the gene expression levels related to osteogenesis (Runx2 transcription factor and alkaline phosphatase (ALP)), 1.5·10^6^ MC3T3-E1 cells were seeded per scaffold and cultured for 7 days (*n* = 3 for each sample). Total RNA was isolated from the cells using a standard procedure (Trizol, Invitrogen, Groningen, the Netherlands), and the gene expression of various osteoblastic markers was analyzed via real-time PCR using a QuantStudio™ 5 real-time PCR system (Applied Biosystems, Foster City, CA), as described by Pérez et al. [34]. Primers for mouse Runx2 and alkaline phosphatase (ALP) were obtained from Assay-by-DesignSM (Applied Biosystems). GAPDH, a housekeeping gene, was amplified in parallel with the target genes. Relative mRNA expression was calculated as 2−ΔCT, where ΔCT = CTtarget − CTGAPDH. For Runx2 (Mm00501578_m1) and ALP (Mm00475834_m1).

#### 2.8.6. Biomineralization Assay (Alizarin Red Staining)

For this assay, three replicates of each scaffold composition were placed in Corning Transwell 12-well plates (Fisher Scientific). Preosteoblastic MC3T3-E1 cell cultures were seeded at a concentration of 1 10^4^ cells/mL directly into each well. Subsequently, the previously sterilized scaffolds were placed in the permeable polycarbonate membrane inserts. The α-MEM medium, with 10% fetal bovine serum (FBS, Gibco, Thermo Fisher Scientific, Wilmington, DE, USA), 1% penicillin-streptomycin, and 5 mM L-glutamine (Gibco, Thermo Fisher Scientific, Wilmington, DE, USA), was changed every third day, and the plates were kept in an incubator at 37 °C with 5% CO_2_ saturation.

Cell mineralization was assessed on the 10th day of incubation using alizarin red staining. To do this, the culture medium and the Transwell inserts containing the scaffolds were removed. The cell-containing wells were rinsed with PBS and fixed in 70% ethanol for 1 h at 4 °C. Subsequently, the cells were stained with 40 mM alizarin red (pH 4.2) for 10–30 min at room temperature. Next, the cells were washed with distilled water to remove nonspecific staining, and the labeling was eluted with 10% (*w*/*v*) cetylpyridinium chloride in a 10 mM sodium phosphate buffer (pH 7). The absorbance of this dilution was measured at 620 nm using a Unicam UV-500 spectrophotometer (ThermoSpectronic, Cambridge, UK).

### 2.9. Statistical Analysis

The results are presented as mean ± standard deviation of at least three independent experiments. Statistical differences were analyzed using ANOVA followed by Tukey post-test with a *p*-value of 0.05, using IBM SPSS statistic 21.0.0.0 version.

## 3. Results

### 3.1. Microstructural Characterization of Bioactive Mesoporous Glasses (MBGs)

**Figure 1:**
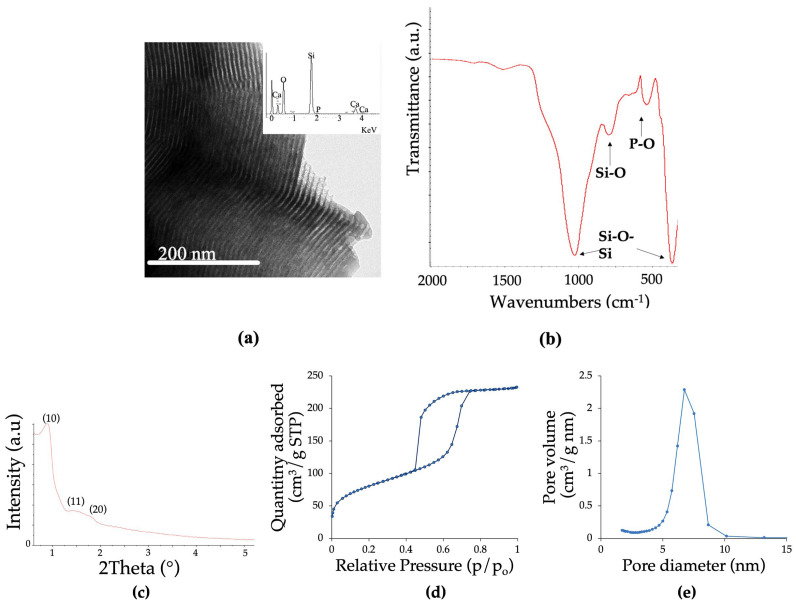
(**a**) Transmission Electron Microscopy (TEM) images and their respective Energy-Dispersive X-ray Spectroscopy (EDS) spectra. (**b**) Fourier-Transform Infrared Spectroscopy (FTIR). MBGs characterization: (**c**) small-angle X-ray diffraction (SA-XRD) patterns, (**d**) nitrogen adsorption–desorption isotherms, and (**e**) pore size distributions.

The results of the characterization conducted using Transmission Electron Microscopy (TEM) and Energy-Dispersive Spectroscopy (EDS), along with Fourier-Transform Infrared Spectroscopy (FTIR), for the synthesized MBGs are depicted in Figure 1a,b. In this figure, we can observe the ordered arrangement of pores in the material, simulating lines or rows oriented in a single direction. Furthermore, the FTIR analysis revealed absorption bands in the ranges of 1040 cm^−1^ and 470 cm^−1^, as well as 800 cm^−1^, respectively, corresponding to the well-established silica network structure found in these types of biomaterials.

Table 1 encompasses the EDX spectra acquired during TEM examinations, thereby substantiating the close resemblance of the MBGs chemical composition to the calculated nominal compositions. Furthermore, this table provides a comprehensive overview of the textural properties of the powders, including BET surface area (S_BET_), pore volume (V_P_), and pore diameter (D_P_), meticulously ascertained through nitrogen adsorption (BET).

In the results of the analyses obtained through Small-Angle X-ray Diffraction (SA-XRD) techniques, we observe well-resolved diffraction maxima at 2θ° = 1.0, which can be attributed to the (1 0) reflection of a p6m 2D hexagonal group. Additionally, there are poorly resolved peaks at around 2.0, which can be assigned to the (11) and (20) reflections, as depicted in Figure 1c.

Furthermore, Figure 1d,e presents the analyses of the textural properties obtained via BET analysis. These figures display the nitrogen adsorption isotherm of the MBGs powders, which exhibits the characteristic Type IV adsorption isotherm of a mesoporous material with a H1-type hysteresis loop. Additionally, the pore size distribution is found to have an average value of approximately 6.6 nm.

### 3.2. Degree of Substitution Analysis of GelMa

**Figure 2:**
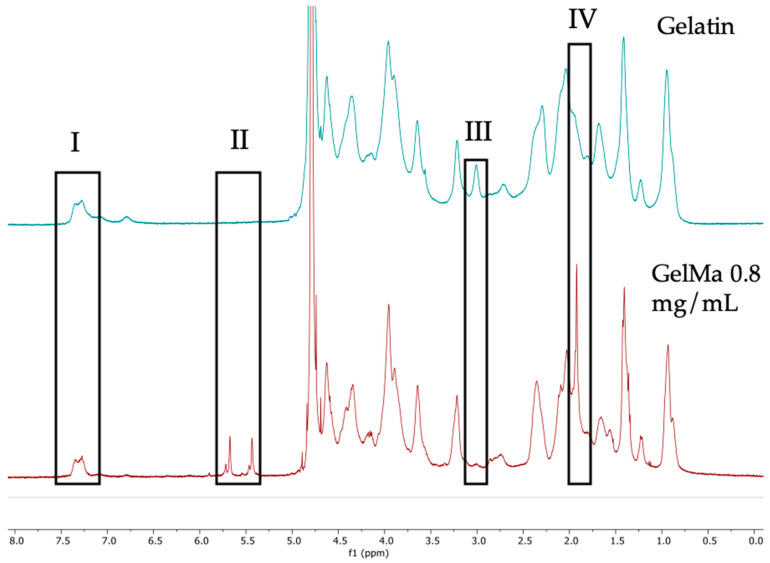
NMR-H spectra of unmodified gelatin and GelMa. Signals: (I) Phenylalanine; (II) Acrylate double bond; (III) Lysine methyl; (IV) Methyl of methacrylate.

Figure 2 displays the 1H NMR spectra corresponding to the gelatin methacrylation reactions (GelMA 0.8 mg/g), along with the spectrum of unmodified gelatin, to calculate the achieved degree of methacrylation. Within the obtained spectra, peaks corresponding to signals from the phenylalanine protons, the methacrylate groups, the methyl group of lysine, and the methyl group of the methacrylates can be observed.

In the spectral analysis, each spectrum was referenced to the 7.4 ppm signal of phenylalanine, an amino acid that remains unchanged during the methacrylation reaction. Using the integral of this signal, the signal at 2.9 ppm of the lysine methyl group was integrated for both the starting gelatin and the methacrylated gelatin. Using this formula, the degree of methacrylation (DM) was calculated, resulting in 84.4%. In other words, the 2.9 ppm signal decreases as the gelatin becomes more methacrylated.

### 3.3. Cytocompatibility Assays of Apigenin, Kaempferol, Extracts, and bLF in Pre-Osteoblastic MC3T3-E1 Cells

**Figure 3:**
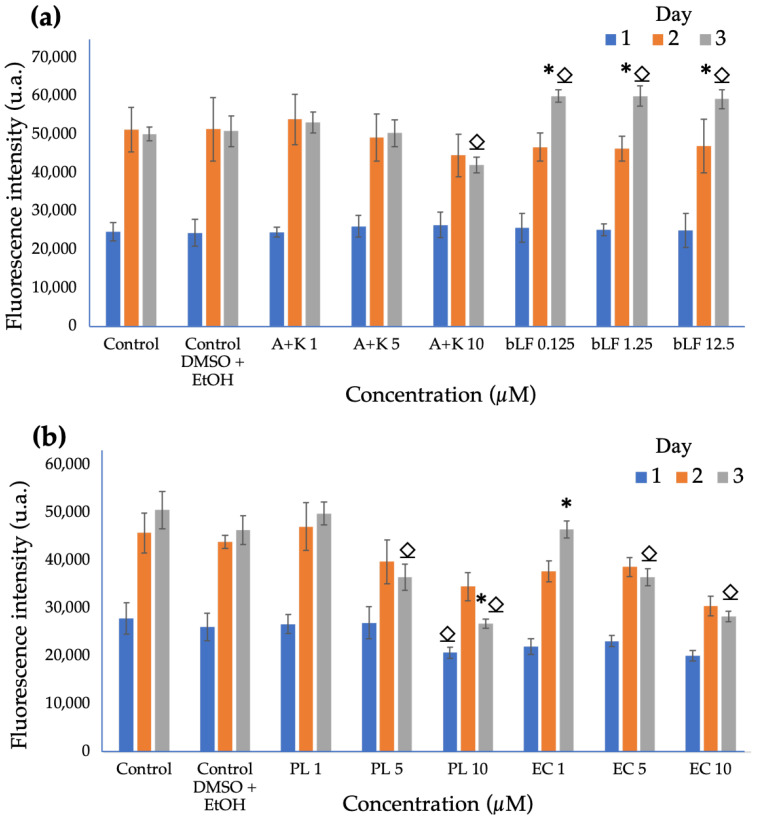
AlamarBlue Cell viability of preosteoblastic cells after 1, 2, and 3 days of culture. (**a**) Apigenin and kaempferol standards (A + K) and bovine lactoferrin (bLF). (**b**) Parsley leaf extracts (PL) and embryogenic cultures (CE). (⍚ = significant differences compared to control, and * = significant differences between cultures at 2 and 3 days of incubation, *p* < 0.05).

The results of the cell viability assay for the standards and extracts are depicted in Figure 3. Samples containing apigenin and kaempferol standards exhibited a marked and proportional decrease in cell viability or proliferation as a function of increasing concentration, and the concentration of 10 µM showed a significant decrease on the third day of cultivation. By contrast, bovine lactoferrin maintained a proliferative activity similar to the control at the lowest concentration, sustaining this behavior and demonstrating a significant difference from the control starting from the third day of cultivation. For this reason, a decision was made to continue using concentrations in the range of 0.094 µM and 0.19 µM in the subsequent tests in order to prevent potential interference with the results.

In contrast to the standards, the extracts, which were loaded based on the quantity of apigenin quantified in each, exhibited a more pronounced relationship between cell viability and concentration. From the first day of cultivation, a significant decrease in cell proliferation was observed at concentrations of 10 µM for PL as compared to the control. On the third day of cultivation, only the concentrations of 5 and 10 µM of the PL and EC extracts showed a significant difference in cell proliferation compared to the control. This result is in agreement with what was observed in the assays with the standards; therefore, subsequent assays were carried out using concentrations based on the quantified amount of apigenin in each extract.

### 3.4. Loading and Release of Extracts in Photopolymerized GelMa/MBGs Discs

Loading analysis was conducted by assessing the changes in porosimetry values exhibited by the MBGs after exposure to standards and extracts and by quantifying these changes using two methodologies. Employing thermogravimetric analysis (TGA), the percentage loading capacity relative to weight was determined. This analysis allowed for the verification of the successful loading of the standard or extract onto the MBGs by detecting mass losses corresponding to the loaded compound.

Furthermore, quantification and supernatant analysis via high-performance liquid chromatography (HPLC) provided the concentrations that the MBGs were capable of absorbing or saturating, as observed in Table 2 and Table 3.

The results of the nitrogen adsorption analysis revealed a significant decrease in both the surface area and pore volume for all loaded MBGs. Concerning the HPLC analysis, the extracts exhibited a loading concentration ranging from 22 to 47 µM (based on apigenin), while the apigenin standard fell within the range of 35 to 57 µM. It is noteworthy that the kaempferol standard exhibited an exceptional loading capacity in the material, reaching a concentration of 49 µM, closely approaching the expected loading concentration of 50 µM.

**Figure 4:**
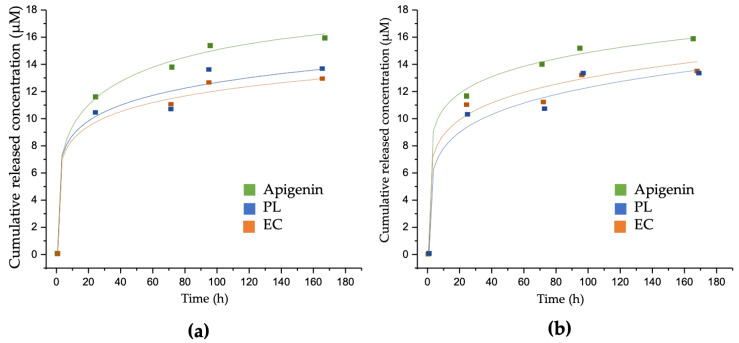
Cumulative release profiles of GelMa/MBGs-loaded discs with apigenin standard and extracts at 7 days of incubation in MilliQ water. (**a**) 50 µM concentrations (**b**) 100 µM concentrations. The indicated points represent the mean of three independently taken values for each time period.

The release kinetics of the standards and extracts from MBGs discs at concentrations of 50 and 100 µM are depicted in Figure 4 and Table 4. The results indicate that, within the initial 24 h, apigenin is released from the material’s surface, suggesting a weak interaction between the compound and the material during this period. However, over the subsequent 144 h, release becomes more challenging. By contrast, kaempferol was not released on any of the days evaluated; therefore, it is not represented in the figure and tables.

The release data for both apigenin standards and extracts were better fitted to a first-order kinetic model with an empirical non-ideality factor (δ). This model takes into consideration the presence of imperfections or heterogeneities in the release system matrix that may affect the compound’s release rate. In all samples, the peak of release was observed within the initial 24 h. Subsequently, the releases obtained remained within the ranges of cumulative concentration that cells can assimilate, ranging between 5 and 20 µM. This substantiates the fact that their release remains biologically active over time.

### 3.5. Cytocompatibility Assays of Meso-Macroporous GelMa/MBGs Scaffolds Loaded with Extracts and bLF

#### 3.5.1. Characterization of Meso-Macroporous 3D GelMa/MBGs Scaffolds

**Figure 5:**
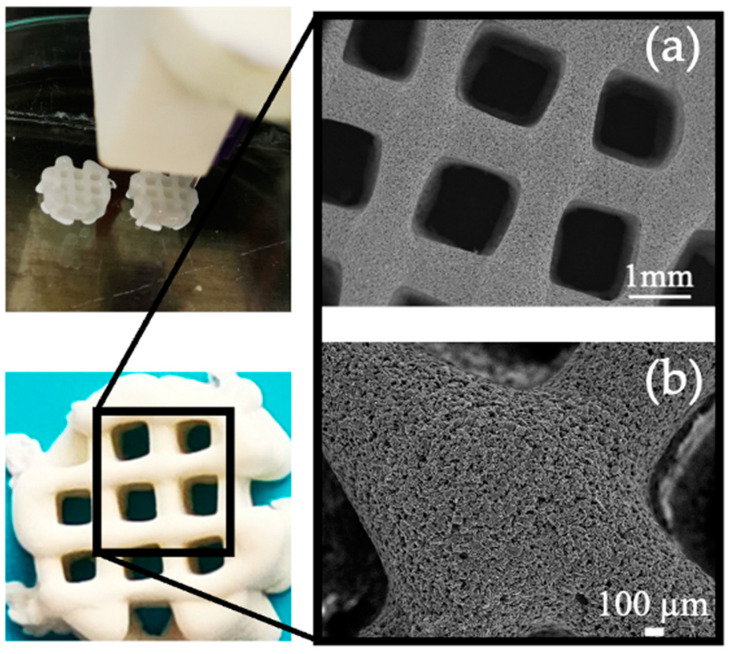
Photographs and scanning electron micrographs obtained from the central view of a MBGs scaffold. In these images, the giant channels (**a**) and macropores (**b**) of the structure are clearly discernible.

The dimensional features of the scaffolds, derived from 3D printing, following the integration of the mixture comprising GelMa, bLF, and MBGs powders, and followed by the photopolymerization process, are illustrated in Figure 5.

**Figure 6:**
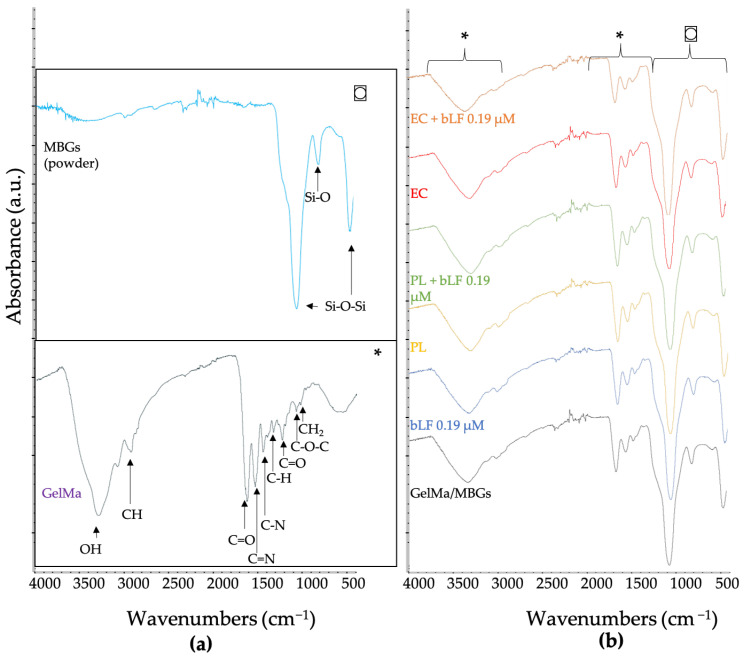
FITR spectra: (**a**) MBGs (powder) and GelMa, (**b**) GelMa/MBGs scaffolds blank and loaded with parsley leaf extracts (PL), embryogenic cultures (EC), and bLF. ⌼ and * magnifications of (**a**).

The peaks detected in white MBGs powder coincided with the spectrum described previously in Section 3.2. GelMa displayed bands at 1243, 1555, 1645, 3063, and 3430 cm^−1^, corresponding to repetitive units known as amides 1, 2, and so forth, as is shown in Figure 6.

Furthermore, the spectra of samples loaded with extracts and bovine lactoferrin (bLF) demonstrate the integration of gelatin and MBGs in each of them. However, due to the intensity of the peaks represented by the glass and GelMa, it is not possible to discern the bonds corresponding to phenolic compounds.

**Figure 7:**
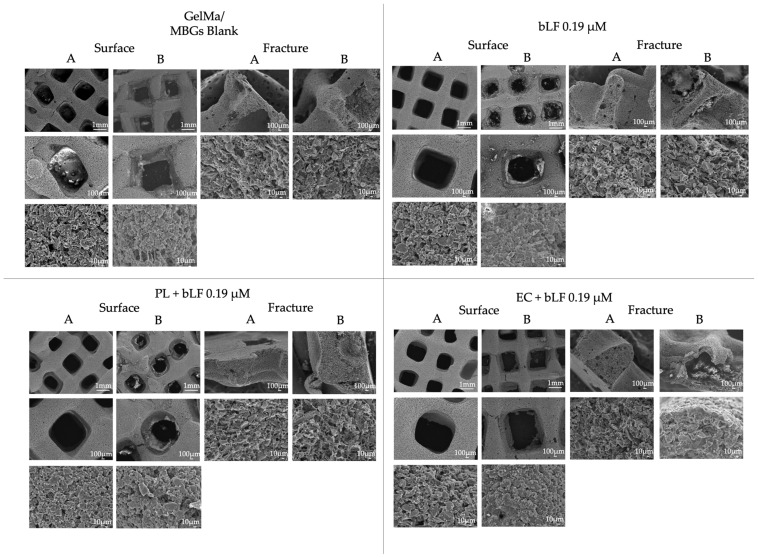
SEM micrographs of the surface and a fracture of GelMa/MBGs blank scaffolds, bLF 0.19 µM, parsley leaf (PL), and embryogenic culture (EC) extracts + bLF 0.19 µM. (**A**) Scaffolds before immersion. (**B**) Scaffolds after being immersed for 10 days in the culture medium.

SEM micrographs of the blank MBGs scaffolds, bLF 0.19 µM, and the mixture of bLF with the extracts before and after being in contact with the culture medium for 7 days are presented in Figure 7. As observed, prior to immersion, all compositions exhibit a well-defined channel structure with sizes ranging between 700 and 1000 µm. However, upon material immersion, partial surface degradation is evident—an expected and characteristic phenomenon resulting from the gradual solubility of GelMa and MBGs.

It is noteworthy that, particularly in the samples utilizing EC extract, a disintegration or hollowing of their structure is noticeable, as seen in the fracture of the EC + bLF 0.19 µM bars (Fracture B). The image clearly depicts the disintegration of the center of the bars, resulting in a completely porous and hollow interior.

#### 3.5.2. Cytocompatibility and Morphological Assays of Meso-Macroporous 3D GelMa/MBGs Scaffolds Loaded with bLF and Extracts

**Figure 8:**
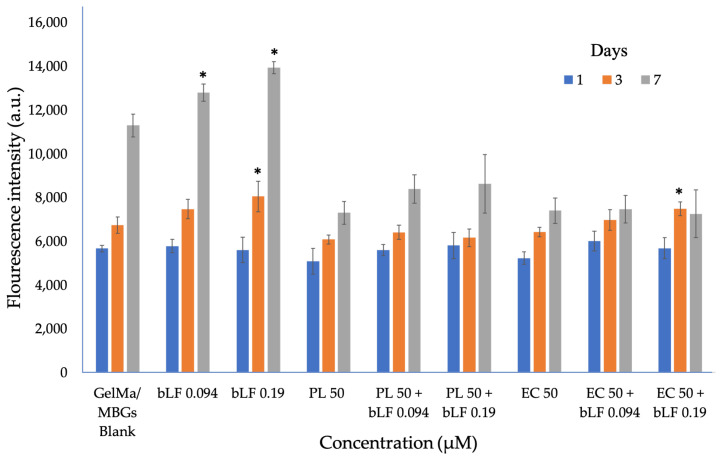
Proliferation of MC3T3-E1 cells on GelMa/MBGs Blank scaffolds and those loaded with parsley leaf (PL), embryogenic culture (EC) extracts, and combined bLF after 1, 3, and 7 days of culture. * = *p* < 0.05 vs. GelMa/MBGs Blank.

The results of the cytocompatibility assay are presented in Figure 8. It was observed that, on the third day of cultivation, samples with bLF at 0.19 µM and EC 50 + bLF at 0.19 µM exhibited significantly different cell proliferation compared to the blank. However, by the seventh day of cultivation, only the samples with bLF at 0.094 and 0.19 µM showed significantly higher proliferation compared to the blank. This suggests that the activity of bLF in cultures has more pronounced effects on cell proliferation over time when used without extracts, as the combination with them limits such proliferation. Therefore, assays such as morphological evaluation or measurement of different parameters like LDH are necessary to determine whether this response is related to cytotoxic effects on cells or other factors.

**Figure 9:**
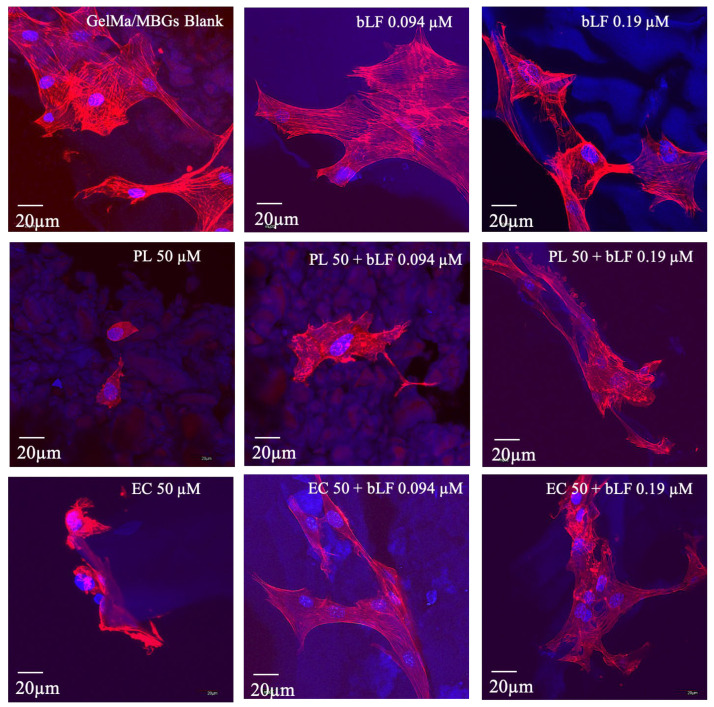
Photographs captured through confocal microscopy depict MC3T3-E1 cells cultivated on GelMa/MBGs Blank scaffolds and loaded with parsley leaf (PL), embryogenic culture (EC) extracts and combined with bLF after 7 days of cultivation. Cellular morphology is revealed through staining with phalloidin, enabling the visualization of actin filaments in the cytoskeleton, while DAPI staining highlights the cell nuclei.

To further extend the analysis of cytocompatibility, an evaluation of the morphology of MC3T3-E1 cells was carried out using confocal microscopy. Scaffolds with 7 days of incubation were selected, and staining with phalloidin (which colors actin filament cytoskeleton) and DAPI (used for nuclear staining) was performed. Figure 9 shows the results obtained from this assessment, revealing that all samples exhibited gradual growth over time. In particular, samples containing PL and EC extracts without lactoferrin demonstrated that the cells were less extended and lacked filopodia (cytoplasmic extensions emerging from the cell membrane) and cellular interconnections.

By contrast, it was observed that all samples containing lactoferrin exhibited typical morphology and displayed optimal colonization and morphology, as well as proper actin cytoskeleton organization.

#### 3.5.3. Lactate Dehydrogenase (LDH) Assay

**Figure 10:**
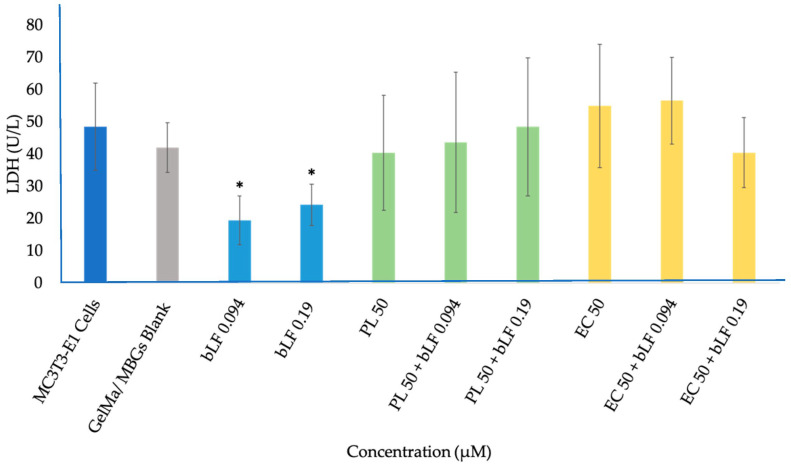
Lactate dehydrogenase (LDH) production by MC3T3-E1 cells cultured on GelMa/MBGs Blank scaffolds and loaded with parsley leaf (PL), embryogenic culture (EC) extracts and combined with bLF after 7 days of incubation. The results are expressed as mean value ± SEM of three measurements for each sample. * Significant difference between extracts and GelMa/MBGs Blank (*p* < 0.05).

The LDH assay results, illustrated in Figure 10, unequivocally demonstrate the absence of cytotoxicity across all samples, with LDH release remaining insignificantly higher than that of the control cells. In simpler terms, none of the samples posed a toxic threat to the cell culture.

Furthermore, only the samples containing bLF demonstrated a significantly lower release of LDH compared to the control and the other samples. These findings corroborate what was observed in the cytocompatibility assay and confocal microscopy, where these samples exhibited enhanced cell adhesion properties and improved cell growth in the cultures.

The results underscore the significance of bLF in promoting cell viability, while the extracts, exhibiting LDH levels similar to the control, demonstrate non-cytotoxicity. In other words, no combination displays cytotoxic effects, suggesting that the reduced viability is likely associated with cellular metabolic activity. Hence, the study of parameters such as PCR and biomineralization becomes crucial for a comprehensive understanding of these processes.

### 3.6. Real Time PCR

**Figure 11:**
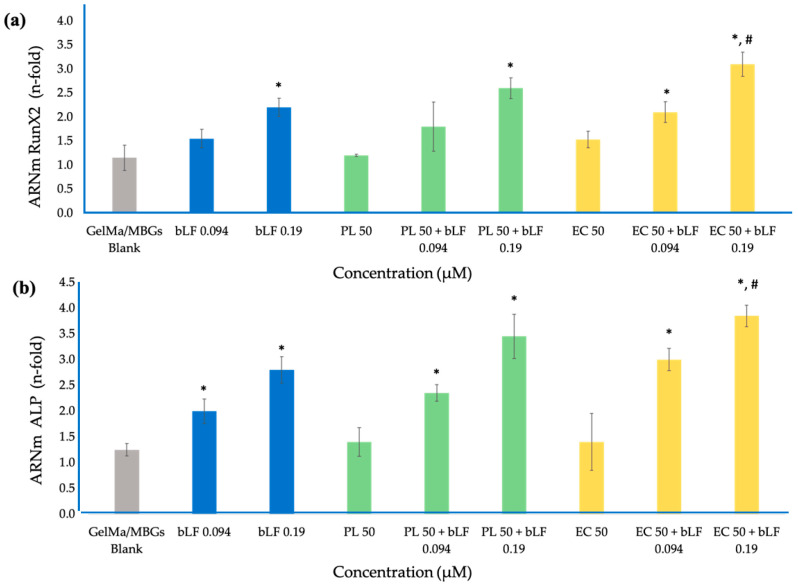
Messenger RNA (mRNA) levels of Runx2 (**a**) and ALP (**b**) genes measured via real-time PCR in MC3T3-E1 cells in the presence of blank MBGs scaffolds and MBGs scaffolds loaded with parsley leaf extracts (HP), embryogenic cultures (CE) and combined with bLF after 7 days of incubation. The results are expressed as mean value ± SEM of three measurements for each sample. * = Significant difference vs. blank MBGs, # = Significant difference vs. bLF (*p* < 0.05).

Figure 11 displays the results obtained from the measurement of Runx2 expression and alkaline phosphatase (ALP) enzyme activity in MC3T3-E1 cells cultured on various scaffolds over a 7-day period.

These results revealed a significant difference in the expression of both factors in all samples containing bLF, regardless of its concentration, compared to the control group. Furthermore, the combination of bLF at 0.19 µM with PL and EC exhibited significantly higher expression compared to the control samples, and only the mixture with CE demonstrated higher expression than the sample containing bLF at 0.19 µM alone. This suggests an additive effect of these combinations that is effective in promoting osteoblastic activity in comparison to the use of the biomaterial alone.

### 3.7. Biomineralization Assay (Alizarin Red Staining)

Alizarin red staining enabled us to qualitatively and quantitatively assess the formation of calcium nodules in the extracellular matrix of MC3T3-E1 cell cultures exposed to different scaffolds over a 10-day period.

**Figure 12:**
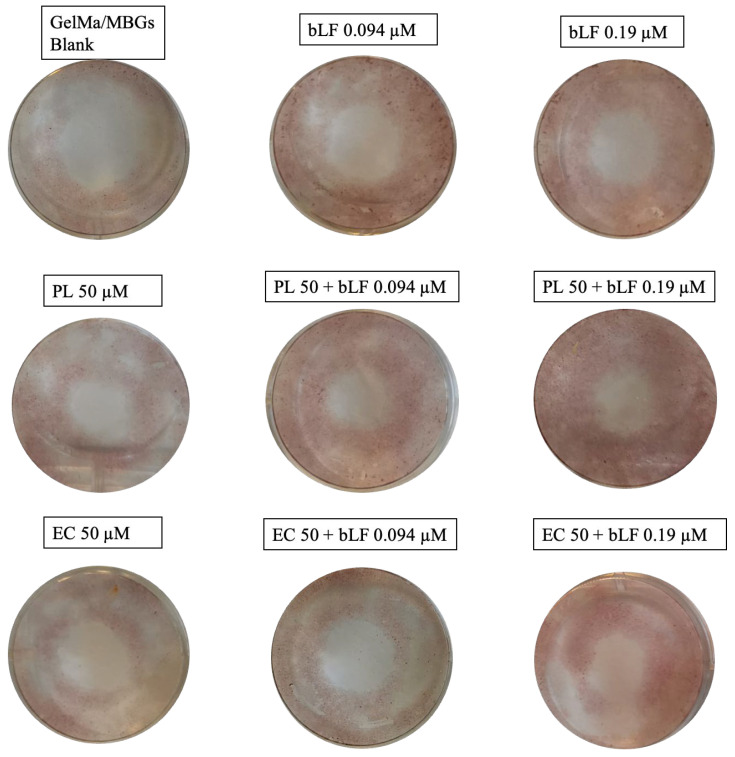
Macroscopic photographs of extracellular matrix mineralization by MC3T3-E1 cells through alizarin red staining in the presence of MBGs scaffolds after 10 days of incubation.

Macroscopic observations revealed visual differences, particularly in samples containing a concentration of 0.19 µM bLF, and notably in the mixture including the PL extract, as illustrated in Figure 12.

**Figure 13:**
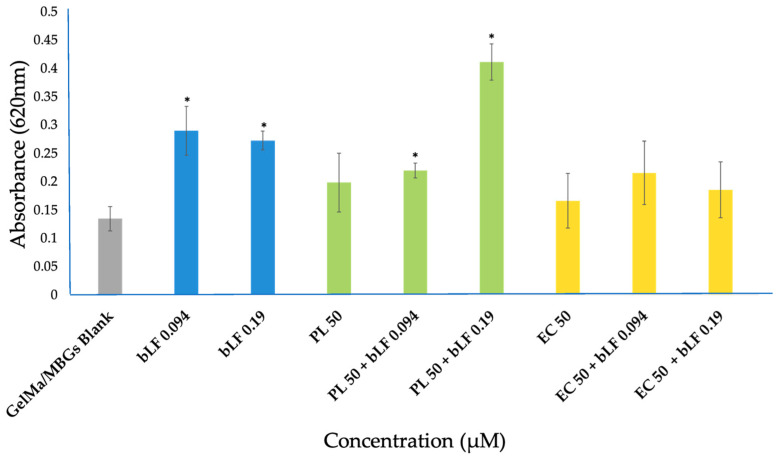
Absorbance of calcium nodules stained with alizarin red in MC3T3-E1 cells cultured in the presence of MBGs scaffolds after 10 days of incubation. Bovine lactoferrin (bLF), parsley leaf (HP) and embryogenic culture (CE). The results are expressed as mean value ± SEM of three measurements for each sample. * Significant difference between extracts and blank MBGs (*p* < 0.05).

This finding was further validated through absorbance measurements and the acquisition of quantitative comparative data, as displayed in Figure 13. Our findings indicate that the addition of bLF at any of the tested concentrations resulted in a significant increase in bone nodule formation, ranging from 1.5 to 2 times greater than the control. The combination of bLF at 0.19 µM with PL extracts exhibited an even more substantial increase in nodule formation, reaching up to three times that of the control.

In contrast to the previously mentioned results, the samples containing EC extract did not exhibit a significant difference compared to the control.

## 4. Discussion

### 4.1. Microstructural Characterization of Bioactive Mesoporous Glasses (MBGs)

The results of the microstructural analyses support the successful production of stable mesoporous materials through the employed synthesis method. The distinct reflection patterns at 10, 11, and 20 in the XRD spectra indicate a mesoporous arrangement—a noteworthy finding, considering the deviation from previously reported syntheses while still retaining the essential characteristics observed in those biomaterials [7].

This discovery is further complemented by the FTIR analysis, which reveals absorption bands at 1040 and 470 cm^−1^ and 800 cm^−1^, corresponding to the asymmetric flexural vibration of Si–O–Si and Si–O bonds, respectively, inherent to the silica network structure, in accordance with prior research [9].

Moreover, the nitrogen adsorption isotherms exhibit typical characteristics of Type IV, indicative of mesoporous materials, and the hysteresis cycle follows a Type H1 pattern, suggesting the presence of porous materials with well-defined cylindrical channels. These features are of paramount importance, as pore morphology directly impacts the potential loading and release capacity of biomaterials [8,35].

### 4.2. Degree of the Substitution Analysis of GelMa

The results of this analysis are of paramount significance, as the degree of methacrylation exerts a substantial influence on the subsequent crosslinking of functionalized gelatin. A higher degree of methacrylation leads to the formation of more densely crosslinked materials through the process of UV-initiated radical photopolymerization.

Gelatin methacrylation is carried out by reacting the amino groups present in the gelatin with methacrylic anhydride (MA) in a basic environment (pH 10, carbonate buffer). In this case, a concentration of 0.8 mg per gram of gelatin was employed, resulting in the production of methacrylated gelatin. This process enables the creation of hydrogels with excellent thermo-stable, physical, and structural properties, as well as rapid gelation kinetics [36].

During this procedure, the amino groups present in the side chains of gelatin are replaced by methacrylate groups. The degree of substitution of these amino groups allows us to obtain varying levels of methacrylation, depending on the amount of methacrylic anhydride used [37].

Although this study did not address variations in synthesis parameters, it is important to note that several variables influence the physicochemical properties of the hydrogel obtained through radical polymerization. These factors include the degree of hydrophilicity, mechanical properties, and enzymatic degradation [27]. Furthermore, other parameters and factors may influence the resulting hydrogel, such as pH, the presence of biocatalysts, and the incorporation of inorganic additives. By optimizing the composition ratios in the mixtures and adjusting the degree of methacrylation, it is possible to modulate both the degree of crosslinking and the kinetics of hydrogel formation. Additionally, this parameter will also impact the precursor solution of the hydrogel to ensure its consistency is suitable for facilitating easy injection in the 3D printing process of scaffolds [38].

### 4.3. Cytocompatibility Assays of Apigenin, Kaempferol, Extracts and bLF in Pre-Osteoblastic MC3T3-E1 Cells

The results obtained for the standards of apigenin and kaempferol align with previous in vitro studies, which provide an explanation for the observed trends in our study. Apigenin, at concentrations of 10 µM, was reported not to affect the morphology of pre-osteoblastic cells, but it significantly inhibits proliferation in MC3T3-E1 osteoblastic cells [28]. On the other hand, kaempferol has demonstrated the ability to stimulate the proliferation, differentiation, and mineralization of murine pre-osteoblastic MC3T3-E1 cells, enhancing alkaline phosphatase activity and calcification at concentrations of 10 µM. However, it is noteworthy that cell viability begins to be affected beyond this concentration [31]. In both cases, the literature suggests that these compounds are more oriented toward cellular differentiation, and their concentrations are limited due to the effect they can have on cell proliferation [39].

Furthermore, the results obtained in this current study indicate that the cellular response to the presence of bLF is consistent with findings from previous research. Specifically, it has been demonstrated that bLF has the capacity to induce the proliferation of MC3T3-E1 osteoblastic cells through the activation of signaling pathways such as ERK, JNK, and p38, along with the stimulation of transcription factors c-Fos and c-Jun, suggesting molecular-level osteogenic activity [33]. Furthermore, bLF has shown high biocompatibility and cell adhesion capabilities, which contribute to promoting cellular proliferation. This would explain the results obtained, even at lower concentrations [40].

In contrast to the results obtained using standards and bLF, plant extracts display a more pronounced relationship between cell viability and concentration. It is essential to note that these extracts consist of a complex mixture of compounds that can lead to synergistic, additive, or antagonistic interactions and activities. Understanding the biological activity will require conducting multiple cell viability analyses, as this response may be influenced by various factors [41]. Consequently, these findings inspire us to explore further research to enhance our understanding of the effects of plant extracts on cell cultures.

The implementation of techniques such as confocal microscopy allows for a detailed visualization of cellular interactions with the extracts, providing valuable insights into the biological activity of these compounds. Additionally, it would be relevant to perform cytotoxicity analyses and evaluate differentiation markers to investigate both the toxicity of the extracts on cells and their influence on the mineralization and cellular differentiation process. The combination of these techniques will enable us to gain a deeper understanding of the underlying molecular mechanisms behind the observed effects of plant extracts on cell cultures in this analysis [42].

### 4.4. Loading and Release of Extracts in Photopolymerized GelMa/MBGs Discs

The loading of samples into the MBGs was unequivocally confirmed through various analyses. Nitrogen adsorption analysis revealed that all loaded MBGs exhibited a noticeable reduction in their specific surface area and pore volume. This observation aligns with prior studies involving these materials and various pharmaceutical compounds, thus affirming their successful loading [7,34].

The percentage values obtained from the TGA analysis and the quantifications from HPLC further corroborate the relationship between loading and concentration within the MBGs, depending on the loaded compound. The percentage loading capacity of all samples remained consistent with the previously reported ranges for other pharmaceutical compounds. Notably, it was within the range of 2.5% to 8%, similar to natural compounds like curcumin [43]. To date, there have been no reports of utilizing complete plant extracts for loading such biomaterials.

Furthermore, it was observed that kaempferol exhibited the highest loading capacity, attributed to its chemical structure and the availability of hydroxyl groups (-OH). These attributes enable greater interaction with the silanol groups on the MBGs surface through electrostatic interactions [2,44]. Additionally, kaempferol did not release during the evaluation period, primarily due to its low solubility in water [45]. This limited solubility prevents its easy release from the material, as its interaction with the glass matrix is stronger than with water, as previously discussed.

On the other hand, the results regarding the loading and release of apigenin and its extracts underscore the fundamental influence of compound purity and chemical structure on loading capacity and concentration within MBGs. Notably, this was validated by the observation that, despite loading the extracts based on apigenin concentration, the loading capacity of this compound consistently decreased in all cases. This indicates competition among various compounds within each extract for surface interaction with the material. Furthermore, these compounds share chemical structures similar to kaempferol, saturating the material and impeding the attainment of high concentrations of apigenin-like structures.

The release data for both apigenin standards and extracts were best described by a first-order kinetic model with an empirical non-ideality factor (δ). This model accounts for the presence of imperfections or heterogeneities within the release matrix that can influence the compound’s release rate. The observed deviations in all the samples suggest the existence of various factors, such as the distortion of mesoporous channels and the release of adsorbed molecules from the network’s external surface, impacting the linear or sustained release of apigenin [7].

The results obtained in this study demonstrate that, despite their diverse and intricate composition, the extracts exhibit a release behavior analogous to that of previously tested pharmaceuticals (antibiotics and osteoinductive compounds) in MBGs [7,8]. This underscores the fact that the release pattern depends not only on the nature of the compound but also on the biomaterial’s inherent characteristics. The promising findings concerning the osteogenic activity of MBGs with this release kinetics suggest their potential for future applications. The continuous research and optimization of MBGs characteristics and loading capacity are essential to maximize their effectiveness in releasing bioactive compounds and enhance their utility in bone regeneration.

### 4.5. Cytocompatibility Assays of Meso-Macroporous GelMa/MBGs Scaffolds Loaded with Extracts and bLF

#### 4.5.1. Characterization of Meso-Macroporous 3D GelMa/MBGs Scaffolds

The peaks detected in the FTIR spectrum of the synthesized GelMa provide conclusive evidence of gelatin functionalization, which is composed of amino acids linked together by amide bonds. Characteristic bands were identified at 1243 cm^−1^, known as Amide I, which is generally associated with the vibration of the N-H bond (also part of the C-N bond). Additionally, the peak at 1555 cm^−1^, referred to as Amide II, is also related to N-H bonds. The presence of Amide III, observed at 1645 cm^−1^, indicates the vibration of C=O bonds. It has been reported that the position and intensity of the band at 1645 cm^−1^ are linked to the secondary structure of gelatin. Furthermore, bands at 3063 and 3430 cm^−1^ are associated with Amides B and A, respectively. The band at 3063 cm^−1^ is related to variations in C-H bonds, while the band at 3430 cm^−1^ reflects the mobility of N-H bonds, characterizing a successful synthesis of GelMa [46].

Although specific bands for phenolic compounds were not detected, it is anticipated that these compounds are present and overlap with characteristic peaks at 3427 cm^−1^ and 3317 cm^−1^ (phenolic O-H bond), 2954 cm^−1^ and 2850 cm^−1^ (C-H bond), and 1613 cm^−1^ (C=O bond), as previously reported [45].

Moreover, SEM micrographs of the scaffolds, particularly in samples using the EC extract, revealed disintegration and collapse. This phenomenon can be attributed to the physicochemical properties of the compound, as a higher quantity of extract was required to reach the necessary concentrations of apigenin, resulting in compound saturation reflected in the color of the loaded MBGs, which exhibited greater intensity.

It is noteworthy that the photopolymerization process with Irgacure 2959 requires UV light in the range of 360 to 400 nm, which is predominantly absorbed by the phenolic compounds present in the extracts used [47,48]. Therefore, this explains why polymerization was only completed in the outermost layer of the scaffolds, leading to reduced structural stability in samples containing this extract. Consequently, the decision was made to increase the photoinitiator percentage to 2% in subsequent experiments.

#### 4.5.2. Cytocompatibility and Morphological Assays of Meso-Macroporous 3D GelMa/MBGs Scaffolds Loaded with bLF and Extracts

In particular, cytocompatibility with confocal analysis shows that the samples containing PL and EC extracts without lactoferrin suggest that the release of compounds from the extracts may interfere with key cellular processes related to cell adaptation, possibly associated with growth factors or cellular proliferation stimulation.

Furthermore, it was observed that all samples containing bLF exhibited a typical morphology, optimal colonization, and proper actin cytoskeleton organization. This cytoskeleton is essential for maintaining cell shape, enabling cell movement, and facilitating intracellular transport [7]. While only the samples containing lactoferrin showed a significant increase in cell proliferation compared to the control, all the samples displayed interconnected cell populations at different depths within the scaffolds, indicating good cell colonization and communication.

These findings align with previous research highlighting lactoferrin’s ability to biofunctionalize material surfaces and its potential for designing functional biomaterials [40]. Therefore, the inclusion of lactoferrin in GelMa/MBGs scaffolds appears to be a promising strategy for developing osteogenic biomaterials. In summary, these results underscore the pivotal role of lactoferrin as a key component in the preparation of loaded MBGs scaffolds capable of positively impacting initial cell adaptation and proliferation. Furthermore, the use of in vitro study models based on specific cell culture techniques is essential for assessing the potential use of implanted materials and circumventing limitations associated with in vivo models [49].

#### 4.5.3. Lactate Dehydrogenase (LDH) Assay

Commencing with the fact that LDH is a stable cytoplasmic enzyme present in all cells, it is rapidly released into the cell culture supernatant when the plasma membrane is damaged. This release is a distinctive feature of cells undergoing apoptosis, necrosis, or other forms of cellular damage [50]. It is worth noting that none of the samples exhibited significant LDH release, implying that none of the evaluated samples were toxic to the cell cultures. Nevertheless, it is noteworthy that samples containing bLF maintained significantly lower levels than the control. This phenomenon can be attributed to the ability of bovine lactoferrin to reduce apoptosis in primary osteoblast cells in response to serum deprivation. Although it is known that LRP1 is a potent cell surface receptor activated by bovine lactoferrin, it has been demonstrated that bovine lactoferrin enhances PI3K/Akt phosphorylation independently from LRP1. It is important to mention that some studies have suggested that Akt phosphorylation might not be necessary for cell survival mediated by lactoferrin, and the exact mechanism by which lactoferrin prevents cell apoptosis remains not fully understood [51].

It is crucial to emphasize that, despite the lack of prior studies using plant extracts loaded into MBGs, the compounds released in this research did not demonstrate cytotoxicity. This finding complements the results of previous assays and suggests that the compounds in the extracts exhibit activity more related to cellular differentiation than proliferation. This aligns with the outcomes of earlier investigations on phenolic compounds like apigenin and kaempferol, which have highlighted their capacity to promote the differentiation and activation of these cells in the process of accelerated mineralization [17,19,31,42,52].

### 4.6. Real Time PCR

In the context of cellular differentiation and bone tissue engineering, the expression of Runx2 in osteoblastic cells emerges as a focal point. This expression pertains to the production and regulation of the critical transcription factor Runx2, which plays an essential role in osteoblastic cell differentiation, initiating a cascade of molecular events that culminate in bone tissue formation. Furthermore, Runx2 influences the regulation of specific bone-related genes such as alkaline phosphatase (ALP) and osteocalcin, which are fundamental for bone mineralization and remodeling [53].

Concurrently, the activity of the enzyme alkaline phosphatase (ALP) stands out as one of the most relevant early markers of osteoblastic differentiation and bone formation. Increased ALP expression is observed when osteoblastic cells commence synthesizing the extracellular matrix, and this enzyme is secreted into the extracellular space. In this location, ALP hydrolyzes organic phosphates, releasing phosphate ions utilized in bone mineralization [54].

This study focuses on compounds of interest, such as bovine lactoferrin (bLF), apigenin, and kaempferol, which have previously been examined individually. However, this study integrates and assesses their combined effects: bLF incorporated into GelMa, and apigenin and kaempferol contained in extracts. These compounds are found to act additively, enhancing cellular differentiation through their distinct mechanisms of action. While bLF activates PKA and p38 signaling pathways independently of its receptor LRP1, apigenin and kaempferol act through estrogen receptors due to their structural similarity. These compounds have demonstrated a reduction in IL-6 and NO production induced by TNF-α in osteoblasts, subsequently increasing alkaline phosphatase (ALP) activity and collagen content in the cells [30,31,42].

In recent years, within the realm of bone tissue engineering, research has revolved around bone growth factors such as BMP-2 and BMP-7 to stimulate cellular differentiation and tissue growth on scaffolds. However, these factors are costly and necessitate specific conditions to maintain their bioactivity. To overcome these challenges, an alternative approach has been proposed based on agents capable of influencing various intracellular signaling pathways for osteoblastic differentiation. Among these, natural agents, whether of protein or plant origin, have proven effective and cost-efficient [32,55,56].

While previous studies have loaded scaffolds with osteogenic agents such as parathyroid hormone-related protein (PTHrP), osteostatin, and some peptides [34], resulting in the increased expression of differentiation markers, no investigations had been conducted involving plant extracts or combinations thereof with bLF. These findings open up new possibilities for loading GelMa/MBGs scaffolds, enabling the incorporation of novel compound mixtures both within the binding polymer and the material’s pores.

### 4.7. Biomineralization Assay (Alizarin Red Staining)

Despite osteogenesis primarily relying on the proliferation and differentiation of osteoblasts, the formation and mineralization of the extracellular matrix are also crucial processes, which necessitate the presence of calcium and phosphate. Bone nodules, which stain a bright red with alizarin red, develop as calcium, and phosphate levels are deposited in the extracellular matrix and become visible around day 14 of differentiation [57].

In our study, we observed the presence of nodules as early as day 10 of incubation in all samples. The addition of bLF at any of the tested concentrations resulted in a significant increase in the formation of bone nodules, between 1.5 to 2 times more than the control group. The combination of bLF at 0.19 µM with PL extracts showed an even greater increase in nodule formation, reaching up to three times that of the control. These findings suggest that the interaction and release of compounds from both bLF and the loaded extract had an additive effect, facilitating osteoblast differentiation and enhancing the formation of mineralized nodules in MC3T3-E1 cell cultures compared to the other samples [41].

In contrast to the aforementioned results, the samples containing EC extract did not show a significant difference compared to the control group. However, there was substantial variability in the data, indicating that the loss of scaffold stability due to a lack of internal polymerization, as mentioned in Section 4.5.1, may have affected this process due to extract saturation and staining. Nevertheless, the addition of bLF to these samples resulted in increased mineralization activity, confirming once again the additive activity of this compound.

Our findings align with previous research that has highlighted bLF ability to modulate adhesion, morphology, proliferation, and mineralization of the bone matrix in osteoblasts, depending on its release rate and presence in the cellular environment. In recent years, this protein has been administered through various routes, and a better response in bone regeneration has been observed when it is loaded and released in situ [32]. Although its additive effect had previously been observed in hydroxyapatite scaffolds, our results pioneer its use in loaded MBGs with plant extracts, and these findings hold promise for the future investigation and development of more effective, safe, and personalized bone therapies.

## 5. Conclusions

The integration of parsley leaf extracts and embryogenic cultures into MBGs, followed by their combination with GelMa and commercial bovine lactoferrin to produce scaffolds through 3D printing and radical photopolymerization, emerges as a promising alternative in the development of biomaterials. These materials exhibit a significant influence on the expression of differentiation factors and mineralization activities in preosteoblastic cells. The results suggest a substantial potential for these scaffolds to enhance efficiency and expedite the bone regeneration process. Additionally, a notable reduction in the concentrations required to induce a response in preosteoblastic cells was observed. Despite these advancements, further in vivo research is needed to optimize the efficacy of these materials and assess their clinical safety in prospective studies.

## Figures and Tables

**Table 1:** MBGs powders’ textural properties (specific surface area—S_BET_, total pore volume—V_P_, and average pore diameter—D_P_) were determined through N_2_ adsorption. The interplanar d_10_ spacing was obtained via X-ray diffraction (XRD), and the calculated unit cell parameters (a_o_) were also determined. The composition in terms of oxide content for each MBGs was established using Energy-Dispersive X-ray Spectroscopy (EDS), with the intended nominal values indicated in brackets.

MBGs Powder				Atomic Composition wt.% (EDS)		
S_BET_ (m^2^/g)	V_p_ (cm^3^/g)	D_p_ (nm)	a_o_	SiO_2_ (80)	P_2_O_5_ (5)	CaO (15)
303	0.39	6.6	11.4	89.6 ± 2.8	2.8 ± 0.15	8.0 ± 1.5

**Table 2:** Nitrogen adsorption porosimetry values of MBGs loaded and unloaded with extracts. PL: parsley leaf; EC: embryogenic cultures; V_P_: total pore volume; D_P_: pore diameter.

	MBGs	PL 50 µM	PL 100 µM	EC 50 µM	EC 100 µM
S_BET_ (m^2^g^−1^)	254.7	244.3	230.9	240.3	235.2
V_p_ (cm^3^g^−1^)	0.34	0.31	0.33	0.32	0.30
D_p_ (nm)	6.6	6.7	6.8	6.5	6.2

**Table 3:** Percentage loading capacity and saturation concentration determined through TGA and HPLC under saturation conditions.

	Apigenin 50 µM	Apigenin 100 µM	Kaempferol 50 µM	PL 50 µM	PL 100 µM	EC 50 µM	EC 100 µM
Loading capacity (%) TGA	5.3 ± 0.8	6.6 ± 0.9	7.2 ± 1.0	4.6 ± 1.0	4.2 ± 1.0	5.8 ± 0.6	5.3 ± 1.0
Concentration in MBGs (µM) HPLC	35.3 ± 0.2	57.1 ± 0.3	49 ± 0.8	22.6 ± 0.1	43.6 ± 0.3	26.3 ± 0.3	46.8 ± 0.4

**Table 4:** Kinetic parameters of apigenin, parsley leaf (PL), and embryogenic culture (EC) extract release from GelMa/MBGs discs at concentrations of 50 and 100 µM. C_0_ (µM): Initial concentration; (C_t_/C_0_) max (%): percentage of loading; K_1_ (×10^3^ h^−1^) (µM/mm^2^.s): constant release rate; δ: kinetic non-ideality factor; R^2^: goodness of fit.

Sample	C_0_ (µM)	(C_t_/C_0_)_max_ (%)	K_1_ (×10^3^ h^−1^) (µM/mm^2^.s)	δ	R^2^
Api 50	35.3 ± 0.2	45.4	6.2 $\times \;10^{-3}\pm 0.06$	0.22 ± 0.02	0.99
PL 50	22.6 ± 0.1	59.9	1.2 $\times \;10^{-3}\pm \;0.09$	0.15 ± 0.65	0.97
EC 50	26.3 ± 0.3	48.8	1.5 × 10^−3^ ± 0.01	0.15 ± 0.01	0.99
Api 100	57.1 ± 0.3	27.9	4 $\times \;10^{-6}\;\pm \;0.07$	0.13 ± 0.58	0.99
PL 100	43.6±0.3	30.1	8 × 10^–6^ ± 0.15	0.19 ± 1.68	0.98
EC 100	46.8±0.4	28.3	6 × 10^–6^ ± 0.14	0.16 ± 1.43	0.98

## Data Availability

Data are contained within the article.
